# The Conserved Lid Tryptophan, W211, Potentiates Thermostability and Thermoactivity in Bacterial Thermoalkalophilic Lipases

**DOI:** 10.1371/journal.pone.0085186

**Published:** 2013-12-31

**Authors:** Emel Timucin, O Ugur Sezerman

**Affiliations:** Biological Sciences and Bioengineering, Faculty of Engineering and Natural Sciences, Sabanci University, Istanbul, Turkey; Consiglio Nazionale delle Ricerche, Italy

## Abstract

We hypothesize that aggregation of thermoalkalophilic lipases could be a thermostability mechanism. The conserved tryptophans (W211, W234) in the lid are of particular interest owing to their previous involvements in aggregation and thermostability mechanisms in many other proteins. The thermoalkalophilic lipase from *Bacillus thermocatenulatus* (BTL2) and its mutants (W211A, W234A) were expressed and purified to homogeneity. We found that, when aggregated, BTL2 is more thermostable than its non-aggregating form, showing that aggregation potentiates thermostability in the thermoalkalophilic lipase. Among the two lid mutants, the W211A lowered aggregation tendency drastically and resulted in a much less thermostable variant of BTL2, which indicated that W211 stabilizes the intermolecular interactions in BTL2 aggregates. Further thermoactivity and CD spectroscopy analyses showed that W211A also led to a strong decrease in the optimal and the melting temperature of BTL2, implying stabilization by W211 also to the intramolecular interactions. The other lid mutant W234A had no effects on these properties. Finally, we analyzed the molecular basis of these experimental findings *in-silico* using the dimer (PDB ID: 1KU0) and the monomer (PDB ID: 2W22) lipase structures. The computational analyses confirmed that W211 stabilized the intermolecular interactions in the dimer lipase and it is critical to the stability of the monomer lipase. Explicitly W211 confers stability to the dimer and the monomer lipase through distinct aromatic interactions with Y273-Y282 and H87-P232 respectively. The insights revealed by this work shed light not only on the mechanism of thermostability and its relation to aggregation but also on the particular role of the conserved lid tryptophan in the thermoalkalophilic lipases.

## Introduction

Lipases (triacylglycerol acylhydrolases, EC 3.1.1.3) are used in various biotechnological applications due to their ability to participate in diverse reactions with distinct substrate specificities [1]. Despite their wide use, non-optimal conditions faced in industrial processes, e.g. high temperatures, can be detrimental to the protein nature of lipases and thus limit their efficiency in biocatalysis [2,3]. Hence, thermostable lipases are one of the current interests in lipase research [4] and of these, bacterial thermoalkalophilic lipases are of great potential for industrial processes due to their ability to work at elevated temperatures [5]. Up till now, various bacterial strains including *Bacillus thermocatenulatus* (BTL2) [6], *Bacillus stearothermophilus* (L1) [7], *Bacillus thermoleovorans* [8] and *Geobacillus zalihae* [9] that produce thermoalkalophilic lipases have been identified. These lipases share 90% sequence homology with each other, and are related to lipases from gram-positive bacteria by about 30% sequence identity [10]. As a result, findings on one member of thermoalkalophilic lipases tend to also be true for the whole group, owing to their high sequence conservation. Among the biochemical features common to all of the thermoalkalophilic lipases, increased thermostability has been known for some time [10], yet few studies have attempted to identify a molecular mechanism for stability in thermoalkalophilic lipases [7,11]. Thus, the molecular basis of how they maintain stability and activity at high temperatures is an open scientific challenge.

Thermostability of proteins is a recurring topic in basic biochemistry and in biotechnology applications. In its simplest meaning, thermostability is the stabilization of protein structure at elevated temperatures, which can be attributed to various factors including charge clusters, networks of hydrogen bonds and packing/hydrophobic interactions [12]. Accordingly a variety of molecular mechanisms were identified for many thermostable proteins, and for each case distinct inter- and/or intra-molecular interactions such as disulfide bridges [13], ionic pairings [14], and hydrophobic interactions [15,16] are linked to protein thermostability. Hitherto, a universal mechanism for thermostability cannot be assigned and the changes which produce it in different cases are subtle and variable [12,17]. In other words, each protein may allocate a different strategy, which reinforces the necessity of delineating each thermostable protein with different rigor. 

The aggregation tendency of bacterial thermoalkalophilic lipases is well-documented [18,19,20,21]. Aggregation-prone proteins display a concentration-dependent time lag during thermal denaturation owing to the intermolecular interactions populated in the aggregates, and such intermolecular interactions during oligomerization/aggregation contributed to thermostability of many proteins [16,17,22,23,24]. From this perspective, aggregation can be one of the means by which thermoalkalophilic lipases become thermostable.

Rua et al. (1997) investigated the aggregation of BTL2 and found a direct relationship between the molecular mass of the lipase aggregates and the increase in activity upon the addition of 1% (w/v) sodium cholate [19]. They commented that cholate breaks BTL2 aggregates and increases the number of available active sites for lipase to reach its maximum solubility and thus activity. They suggested that aggregation hinders the active site of this lipase, a phenomenon previously observed for other lipases [25]. To us more importantly, their particular observation supports that the BTL2 aggregates occupy natively folded lipases, such that the addition of cholate dissolves the BTL2 aggregates and leaves lipases in their functional form. As a result, the aggregated BTL2, when dissolved by cholate, is able restore its activity, suggesting that the molecular assembly in BTL2 aggregates would be true biochemically defined oligomerizations. In line with this notion, the intermolecular interactions that build up in oligomers/aggregates would contribute to thermostability without affecting the function [22,26]. Therefore we hypothesize that inducing aggregation would potentiate thermostability of BTL2 with respect to the non-aggregating condition. Indeed, a plausible relationship between aggregation and thermostability has already been hypothesized in the same study of Rua et al. [19], though there have not been any conclusive results on this subject. Yet any of the intermolecular interactions leading aggregation have been identified. Identification of the critical residues responsible for such intermolecular interactions is a key to the efforts to decipher not only the underlying forces in aggregation, but also the impact of aggregation on thermostability. 

Rua et al. also investigated the dependency of aggregation on temperature, in which breaking of BTL2 aggregates resulted in a significant activity increase at low temperatures, while it led to only a marginal increase in activity at high temperatures [19]. Accordingly another study also showed that the solubility of BTL2 was increased at high temperatures [18], confirming an inverse relationship between aggregation tendency and temperature. In particular at elevated temperatures the intermolecular attractions loosen and the BTL2 aggregates start to dissolve, whereas the interactions become stronger and aggregates are accumulated at low temperatures, suggesting a temperature-mediated control of the intermolecular interactions during aggregation. Temperature-mediated control of protein interactions is commonly observed for many protein activities [7,27,28,29]. In all of these examples, hydrophobic amino acids play crucial roles in temperature-mediated control via hydrophobic interactions. Such interactions can be reversibly dissociated by temperature switches, in which the increased kinetic energy of hydrophobic amino acids obstructs formation of strong interactions [7,30]. Parallel to the previous examples and taken together with the relatively high hydrophobic amino acid content in lipases [6], the intermolecular attractions in aggregation of thermoalkalophilic lipases would likely to be hydrophobic in nature. Due to strong hydrophobic interactions between lipase monomers, the aggregates may not be dissolved at low temperatures; while increased kinetic energy would lead such interactions to be dissociated and aggregates to be dissolved. In this view, our article intuitively investigates possible hydrophobic amino acids that would induce oligomerization/aggregation and thus would confer thermostability in thermoalkalophilic lipases.

The lid of the thermoalkalophilic lipases which is fully conserved among all of the identified members is composed of about 60 amino acids folded into two surface helices at the entrance of the catalytic cleft [7,31]. Essentially the location of lid on the lipase surface capacitates it to contain amino acids critical to the intermolecular interactions [7,32,33]. Because aggregation renders the active site of the thermoalkalophilic lipase inaccessible to substrate [19], the amino acids that would induce oligomerization/aggregation might be found in the lid owing to its location at the entrance of the active site, while other surface residues would less likely to change the accessibility of the active site. The lid of thermoalkalophilic lipases is also rich in hydrophobic amino acids that will be of interest owing to their substantial roles in temperature-mediated processes. Thus we concentrated our investigations on the hydrophobic amino acids in the lid. Among these hydrophobic amino acids, two tryptophans (W211 and W234) had the highest potential to form strong hydrophobic interactions with their surroundings, which can be attenuated by temperature switches. Accordingly tryptophans -when exposed- can serve as "sticky" sites to induce aggregation [34,35] and can also potentiate thermostability in other proteins [36,37,38]. From this overall perspective, the lid tryptophans (W211 and W234) of the thermoalkalophilic lipases might induce aggregation via hydrophobic interactions that would contribute to thermostability. 

Here, we report biochemical analyses of the wild-type BTL2 and the mutants (W211A and W234A) to reveal the relationship between aggregation and thermostability, and the particular roles of the lid tryptophans in this relationship. The structural impact of the W211A mutation on aggregation was further analyzed in molecular dynamics simulations of the L1 lipase (PDB ID: 1KU0) that was captured in dimeric form. The insights revealed by the computational analysis shed light on the molecular machinery of aggregation as a strategy for thermostability in the thermoalkalophilic lipases.

## Materials and Methods

### Site-directed mutagenesis, expression and purification

Site-directed mutagenesis with overlap extension polymerase chain reaction was performed as explained [39], using the forward; cgattttaagctcgaccaagcggggctgcgccgccagc, the reverse primer; cgattttaagctcgaccaagcggggctgcgccgccagc for W211A and the forward; ggctcaaacgatcccctgttgcgacgtcgacggatactg and the reverse primer; cagtatccgtcgacgtcgcaacaggggatcgtttgagcc for W234A substitutions. Mutations were confirmed by DNA sequencing. The lipases were expressed in *Escherichia coli* SHuffle^®^ Express (New England Biolabs, NEB) cells as described previously in [39]. Affinity purifications were carried out using HisTrap HP columns (GE Healthcare Life Sciences) by the availability of N-terminal polyhistidine tag in all lipases. The quantities of the purified lipases were measured in Bradford protein assays [40] and sodium dodecyl sulfate polyacrylamide electrophoresis (SDS-PAGE) was used to test the purity of the lipases (Figure S1). 

### Lipase activity assays

The lipase activity was measured using 4-methylumberrylferyl (4-MU) caprylate as substrate in 100 mM Tris-Cl at pH 7.0. Gemini XS (Molecular Devices) was used to measure 4-MU fluorescence using an excitation wavelength of 355 nm and an emission wavelength of 460 nm in a kinetic fashion. Thermoactivity assays were performed by measuring the real-time lipase activity in the temperature range of 40-90°C and percent hydrolysis was calculated by setting the highest activity to 100%. Thermostability assays were performed by screening the residual lipase activity of the samples that were incubated in the temperature range of 45-90°C for 30 min. Different lipase concentrations were tested during thermal incubation and the reactions were carried out at room temperature. The reaction rates without thermal incubation were set to 100%. For both of thermoactivity and thermostability assays, the lipase concentration in the reaction mixture was set to 10 nm and the assays were repeated two times to test for reproducibility. 

### Dynamic light scattering

Dynamic light scattering (DLS) was conducted using Zetasizer Nano XS (Malvern Instruments) at 25°C. Samples containing 2, 4, 10 or 12 µM of lipases in 5 mM Tris-Cl pH 7.0 were filtered (0.22 µm) and analyzed in DLS. The measurements were repeated three times. The hydrodynamic radius (*R*_*H*_) and the standard deviation of the radius (*C*_*P*_) were collected from monomodal data using the Zetasizer software and percent *C_P_/R*_*H*_ was used to determine the polydispersity index (PDI). 

#### ANS fluorescence

Fluorometry was performed according to the described procedure [41]. In this procedure 1-anilinonaphthalene-8-sulfonate (ANS) was used as fluorescent probe at the excitation wavelength of 360 nm and the emission of 460 nm for two solutions; one with the lipases (50 µM in 5 mM Tris-Cl at pH 7.0), one with both lipases and 5% 2-propanol. These solutions were incubated at the corresponding temperatures without ANS and then incubated with 50 µM ANS for 10 minutes before readings. The measurements were normalized to the background ANS fluorescence from samples without protein and were conducted in duplicates. 

### Circular dichorism spectroscopy

Far-UV circular dichorism (CD) spectra were collected using J-815 spectropolarimeter (Jasco) in N_2_ atmosphere equipped with thermostatically controlled cuvette with 1.0 mm path length at a scanning speed of 100 nm/min. Three scans were averaged to obtain final spectra of 25 µM of wild-type and mutant lipases in 5 mM Tris buffer (pH 7.0), which was corrected for the background. Mean residue ellipticity [θ] is calculated from the equation,


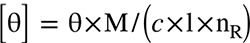


*M* is the molecular mass in g/mol and *c* is the concentration in mg/ml, *l* is the cell length in centimeters, and *n*_*R*_ is the number of residues. The thermal denaturation profiles were determined by tracing ellipticity at 222 nm at a 5°C/min heating rate from 30°C to 90°C and the *T*_*m*_ values were calculated from the midpoint of the transition curves between folded and unfolded states of the lipases.

### Molecular dynamics simulations

Molecular dynamics (MD) simulations were performed using the closed structure of the *Bacillus stearothermophilus* L1 lipase (PDB ID: 1KU0) [7] that shares 90% of sequence homology with BTL2. The W211A mutant dimer was generated via *in-silico* substitution of W211 to alanine in both of the chains. The experimental tests indicated that W234A yielded similar results with the wild-type lipase. Therefore, the MD simulations were carried out only for W211A mutation to address a molecular basis for the loss of aggregation tendency in this mutant. 

Wild-type and mutant dimer systems that composed of ~66,000 atoms were placed in a water box with dimensions of 73x75x121 Å^3^. The systems were generated after solvation with a 100 mM NaCl solution and used in MD simulations using the NAMD program [42] with the CHARMM22 parameters [43,44] which included correction map (CMAP) for backbone atoms [45,46]. Water molecules within the system were treated explicitly using the TIP3P model [47]. An NpT ensemble was used in MD simulations with periodic boundary conditions, and the long-range Coulomb interactions were computed using the particle-mesh Ewald algorithm. Pressure was maintained at 1 atm, at different temperatures of 25°C, 75°C and 125°C using the Langevin pressure and temperature coupling. A time step of 2 fs was used in all MD simulations. Following a 10,000-step energy minimization, the equilibration simulations lasted 10 ns and were repeated twice. Visual molecular dynamics (VMD) [48] was used for the analysis of trajectories and the visualization of structures. Root mean square displacements (RMSD) for the backbone atoms (C, N, Cα) and residue-wise root mean square fluctuations (RMSF) of Cα atoms were measured and shown in supporting information (Figures S5-S6). Percent hydrogen bond occupancies were determined with the threshold of 3 Å for H--O distance and the cutoff of 20° for RHO angle.

### FoldX calculations

Protein design tool FoldX (version 3b51) [49,50] was used to assess the impact of mutations on the lipase stability. The aromatic cluster residues (Y273, Y282 and W211) in the equilibrated dimer structure (1KU0) were individually mutated to three aromatic hydrophobic residues (F, Y and W) and alanine to reveal the impact of the aromatic cluster on the intermolecular interactions. During calculations, the temperature was set to 298K and average of five runs was used to compute the ΔΔG. The changes in the stability (ΔΔG) of mutants were calculated by comparing the free energies of mutant structures with that of wild type. FoldX was also used to analyze the impact of W211 residue on the monomer stability. For this, monomer lipase corresponding to the structure with PDB ID: 2W22 and with chain A of 1KU0 was mutated to W211A, W211F and W211Y and the stability change was calculated by FoldX similarly. 

## Results

### The effect of concentration on aggregation tendency

The predominant oligomeric form of the BTL2 and the mutants in solution were analyzed by dynamic light scattering (DLS) at 25°C. The results of three measurements are summarized in Table 1, and the size-intensity and the size-volume distributions were plotted in Figure S2. For the multimodal data, the peaks that have the largest area in volume distributions were chosen to comment on the majority of the particles.

**Table 1:** Dynamic light-scattering results.

	***C* (µM)**	***R*_*H*_ (nm)**	***C*_*P*_ (nm)**	**PDI**	**%Volume**
**BTL2**	2	13.8±1.3	1.9±0.9	13.5	77.3±2.5
	4	11.6±0.3	1.2±0.0	10.2	84.7±1.2
	10	38.5±1.0	11.0±1.1	28.4	97.3±1.3
	12	40.0±6.6	21.2±17.9	52.9	60.1±2.9
**W211A**	2	3.9±0.2	0.2±0.0	6.3	98.7±0.6
	4	4.4±0.2	0.1±0.0	2.7	99.7±0.3
	10	6.7±0.7	0.8±0.1	11.7	87.4±2.0
	12	6.0±0.2	0.6±0.0	9.4	96.5±0.6
**W234A**	12	66.7±36.5	25.4±18.4	38.0	74.0±11.7

Mean±SD of 3 measurements are given where *C* is the concentration, *R*_*H*_ is the hydrodynamic radius, *C*_*P*_ is standard deviation in the radius, and PDI (*%C_P_/R*_*H*_) corresponds to the polydispersity index.

The size distribution of BTL2 showed that the dominating particles had a hydrodynamic radius around 12 nm at 2 and 4 µM and around 40 nm at 10 and 12 µM (Table 1), which are all larger than a monomer with the hydrodynamic radius of 3 nm [18]. Nevertheless there was a clear distinction between the low (2, 4 µM) and the high (10, 12 µM) concentrations by means of dispersity. Specifically, the BTL2 peaks in radius distribution are more defined indicating low polydispersity (<15%) at low concentrations, while the lipase showed polydispersity with higher PDI values (>15%) at high concentrations, which is an indication of aggregation (Table 1 and Figure S2).

For the lid mutant W211A, DLS measurements showed low PDI values at all of the concentrations, indicating monodispersity in the W211A regardless of concentration (Table 1). When the concentration was as low as 2 or 4 µM, the W211A mutant displayed monomeric lipase at the radius of 4 nm [18]. When the concentration is increased to 10 or 12 µM, the dominating particles in the W211A samples had the radius of 7 nm (Table 1). At high concentrations, the rest of the particles that correspond to about 10% of the W211A mutant are similar in size and shape to the ones observed for aggregated BTL2 (Figure S2), indicating that a small portion of the W211A mutant was still aggregating at high concentrations. These results suggest the aggregation tendency of BTL2 was substantially, but not completely, compromised by the W211A mutation. Despite the fact that the W211A mutation minimized aggregation tendency of BTL2, the other lid mutant W234A showed comparable size-distributions with BTL2, such that it had high PDI value (38%) at 12 µM (Table 1), indicating aggregation similar to BTL2. Overall, DLS measurements showed that the aggregation of BTL2 is induced at high concentrations, and the lid tryptophan W211A mutation impairs aggregation tendency of BTL2, while the W234A does not.

### The effect of temperature on aggregation

**Figure 1:**
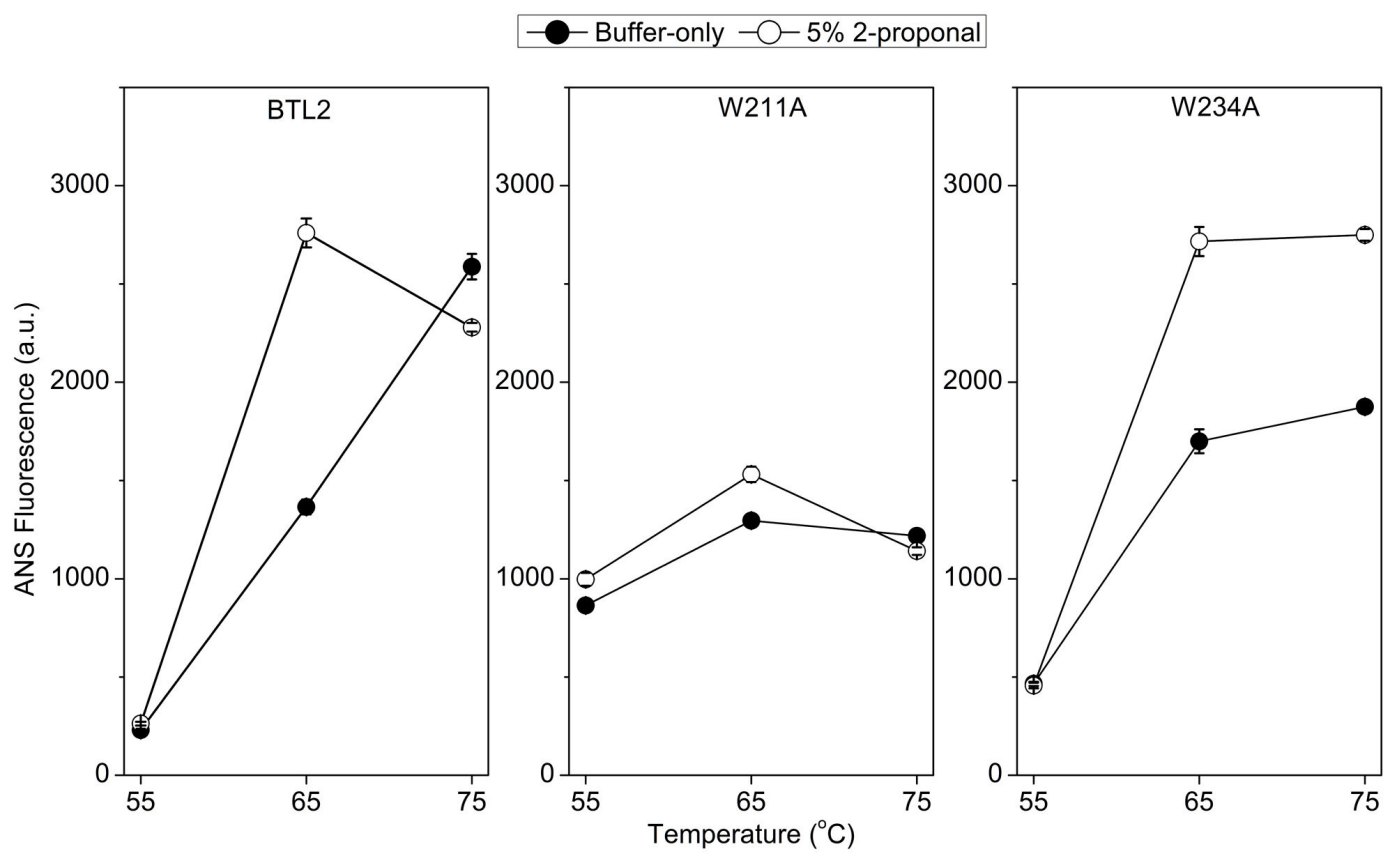
The ANS fluorescence at 460 nm. The closed circles show the fluorescence from the lipases in 5 mM Tris-Cl at pH 7.0 and the open circles show the effect of 2-propanol.

To see the impact of aggregation on the ANS fluorescence, we eliminated the aggregation tendency by dissolving the lipases in 5% of 2-propanol which blocks aggregation of BTL2 [18] (Figure 1). For BTL2, the presence of 2-propanol increased the ANS fluorescence, implying that eliminating aggregation has resulted in a increase in the fluorescence. In this context, the higher ANS fluorescence would indicate the lower aggregation tendency. 

For W234A, the presence of 2-propanol decreased the aggregation tendency, similar to the BTL2. Moreover, both lipases showed higher ANS fluorescence at 65°C than that at 55°C, suggesting that aggregation is decreased by high temperatures for the BTL2 and the W234 mutant. On the other hand, the W211A mutant gave a fairly different profile from BTL2 and W234A, such that ANS fluorescence was merely changed by temperature in the W211A mutant. Specifically, at 55°C the W211A mutant gave the highest ANS fluorescence which can only be reached by BTL2 and W234A at 65°C. Besides, 2-propanol was ineffective to alter the ANS signals received from W211A regardless of temperature, indicating that the W211A mutant lowered the aggregation tendency of BTL2. Overall, ANS binding assays reflected that aggregation is mediated by temperature switches in BTL2, which is conserved by W234A, but altered by W211A mutation. 

### The effect of aggregation on thermostability

**Figure 2:**
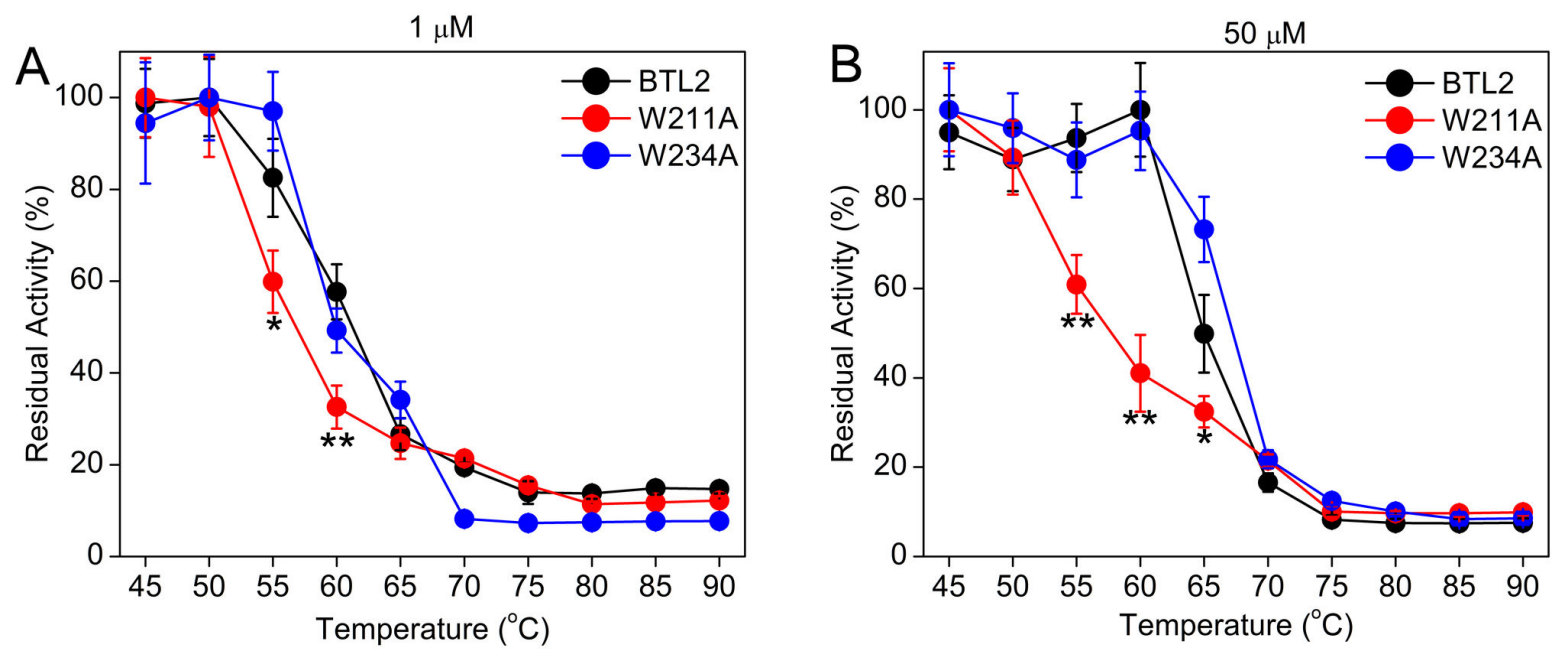
Thermostability at different incubation concentrations. Residual lipase activity after thermal incubation for 30 minutes at concentrations of (A) 1 µM and (B) 50 µM. Student's t-test were performed to determine the significant differences in thermostability of W211A with respect to BTL2 (*p=0.1 and **p=0.05).

To elucidate the effects of aggregation on thermostability, Figure 2A and 2B show the residual lipase activity after thermal incubation at 1 µM and 50 µM concentrations respectively. According to the DLS analyses (Table 1), the concentration of 1 µM indicated monodispersity, while 50 µM of concentration would indicate the condition that favors aggregation for BTL2.

When the thermal incubation was performed at 1 µM, the thermostability of BTL2 agreed with the previous results [19] such that it restored 60% activity at 60°C, which reduced to 30% at 65°C (Figure 2A). When the incubation concentration was set to 20 µM or 50 µM, it was shown that BTL2 restored an activity of 95% at 60°C and of 50% at 65°C (Figure 2B and S3), suggesting a boost in thermostability compared with that for the incubation at 1 µM. These residual activity profiles reflected the fact that the thermostability of BTL2 is improved when the lipase was aggregated during thermal incubation. 

For the lid mutants, we observed that the W211A led to a decrease in the thermostability of BTL2 as it exhibited an activity of 30% at 60°C and 25% at 65°C under non-aggregating condition (1 µM) (Figure 2A). When thermal incubation was performed at 50 µM (Figure 2B), in contrast to BTL2, the W211A mutant has failed to enhance the thermostability for the temperature range of 60-65°C. On the other hand, the W234A displayed 50% activity at 60°C and 34% activity at 65°C when incubated at 1 µM (Figure 2A), and when the lipase concentration was increased to 50 µM, it showed an improvement in the thermostability similar to BTL2, such that it displayed an activity of 95% at 60°C and 50% activity at 65°C (Figure 2B). Taking into account all of the thermostability screens (Figures 2 and S3), the BTL2 and W234A improve thermostability in the temperature range of 60-65°C when incubated at high concentrations that promote aggregation, while W211A reduces thermostability of BTL2 regardless of incubation concentration. 

#### Determination of thermoactivity

**Figure 3:**
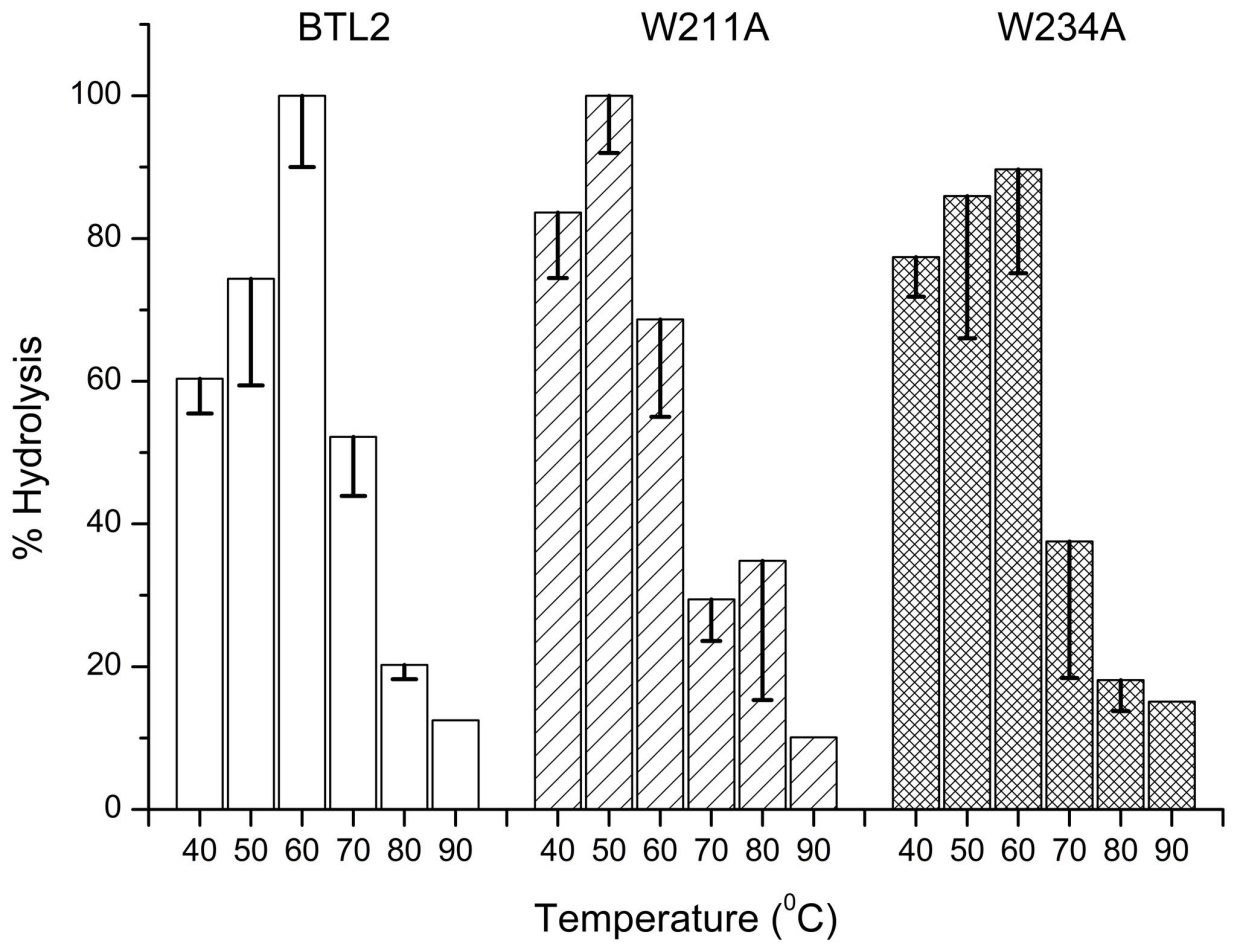
Thermoactivity profiles. Percent lipase activity as a function of temperature is shown in the temperature range of 40-80°C with the error bars for the deviations from two independent assays.

Temperature activity assays were performed to determine the optimal temperature (*T*_*opt*_) for lipase activity. Figure 3 shows that *T*_*opt*_ is 50°C for W211A and is 60°C for BTL2 and W234A mutant, suggesting a significant decrease in the *T*_*opt*_ of W211A mutant yet an indifferent *T*_*opt*_ for W234A mutant. To make a clear comparison of *T*_*opt*_ of BTL2 and W234A, we also investigated the temperature range of 50-70°C by 2°C steps and found that BTL2 reached a maximum activity at 62°C, whereas that of W234A was at 58°C (Figure S4). 

### Determination of thermal denaturation profiles

**Figure 4:**
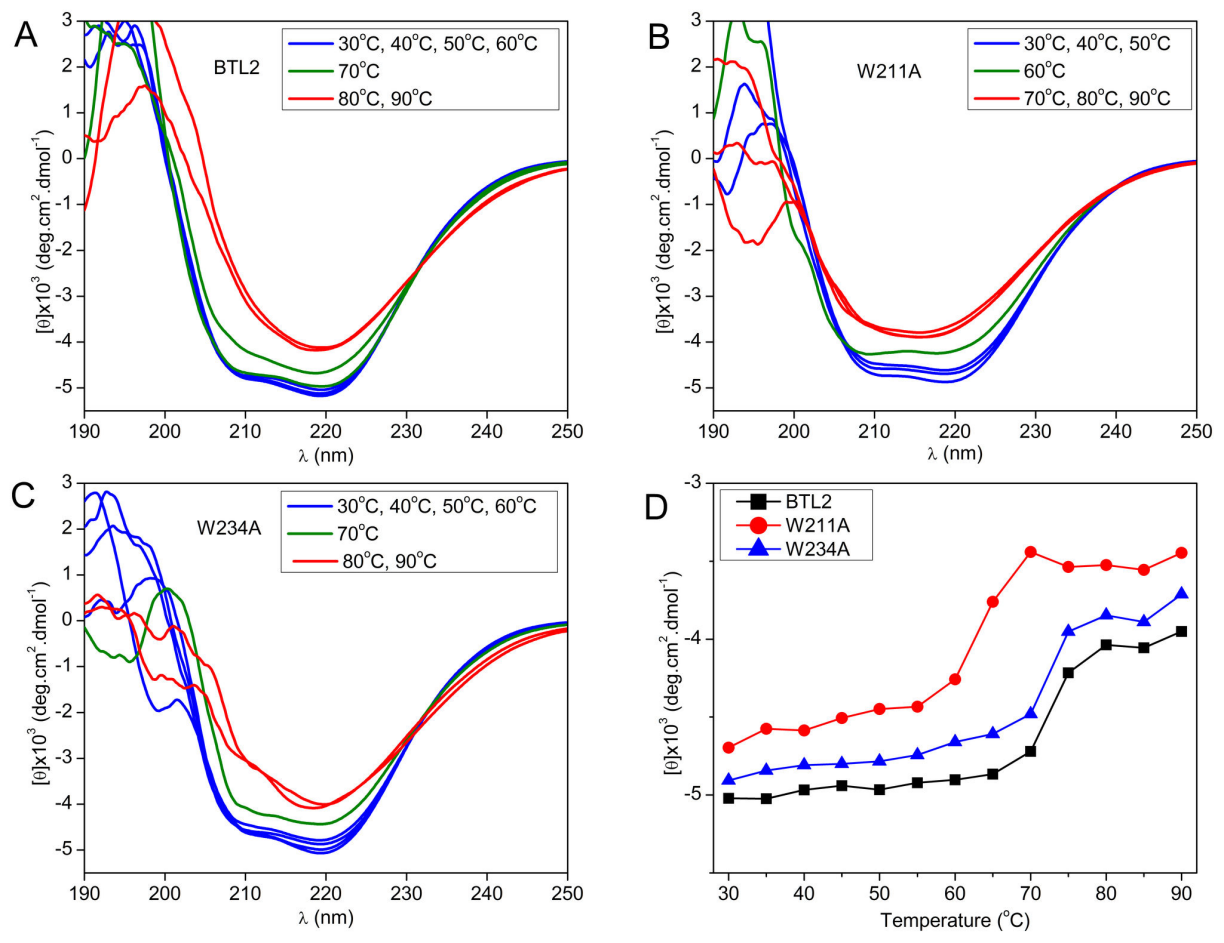
CD-spectra and thermal denaturation. The far UV CD-spectra collected at various temperatures are shown for the lipases (A) BTL2, (B) W211A, (C) W234A, and (D) mean residue ellipticity at 222 nm is plotted as a function of temperature.

CD spectroscopy was performed to monitor the structural stability of all lipases and Figure 4 shows the far-UV CD spectra collected at various temperatures. The CD signal around 205-230 nm did not differ from BTL2 to the W234A mutant, such that both lipases exhibited fairly stable conformation in the temperature range of 30-60°C and experienced a temperature induced change at 70°C (Figure 4A-C). However, the mutant W211A showed a stable structure in the range of 30-50°C, while it displayed an apparent change at 60°C (Figure 4B). For the temperature range of 80-90°C, all three lipases showed drastic decrease in the CD signal around 200-230 nm, which was an indication of dramatic structural changes e.g. unfolding. 

The CD signal at 222 nm corresponding to the alpha-helical content can be related to folded state and here it was recorded over a range of temperature to profile thermal denaturation (Figure 4D). During temperature-induced denaturation, flattening of the ellipticity at 222 nm was distinguishable from the Figure 4D, suggesting a two-state unfolding for all of the lipases. The melting temperature (*T*_*m*_) for BTL2 was found to be 72.3°C, which did not change for the W234A mutant (72.0°C), but decreased significantly for the W211A mutant (63.8°C). The *T*_*m*_ results revealed that W211 destabilized the lipase structure at high temperatures whereas W234 did not affect the thermal denaturation of BTL2. The thermal denaturation profiles were parallel with trends in the *T*_*opt*_ (Figure 3), in which W211A mutation resulted in a notable decrease (~10°C) in the *T*_*m*_ and the *T*_*opt*_ of the BTL2 (Figure 4D), implying that W211 is essential residue for the stability of catalytic machinery and overall structure of BTL2 at high temperatures. However, W234 does not have any significant impacts on such properties. 

### Structural investigations of the thermoalkalophilic lipases

**Figure 5:**
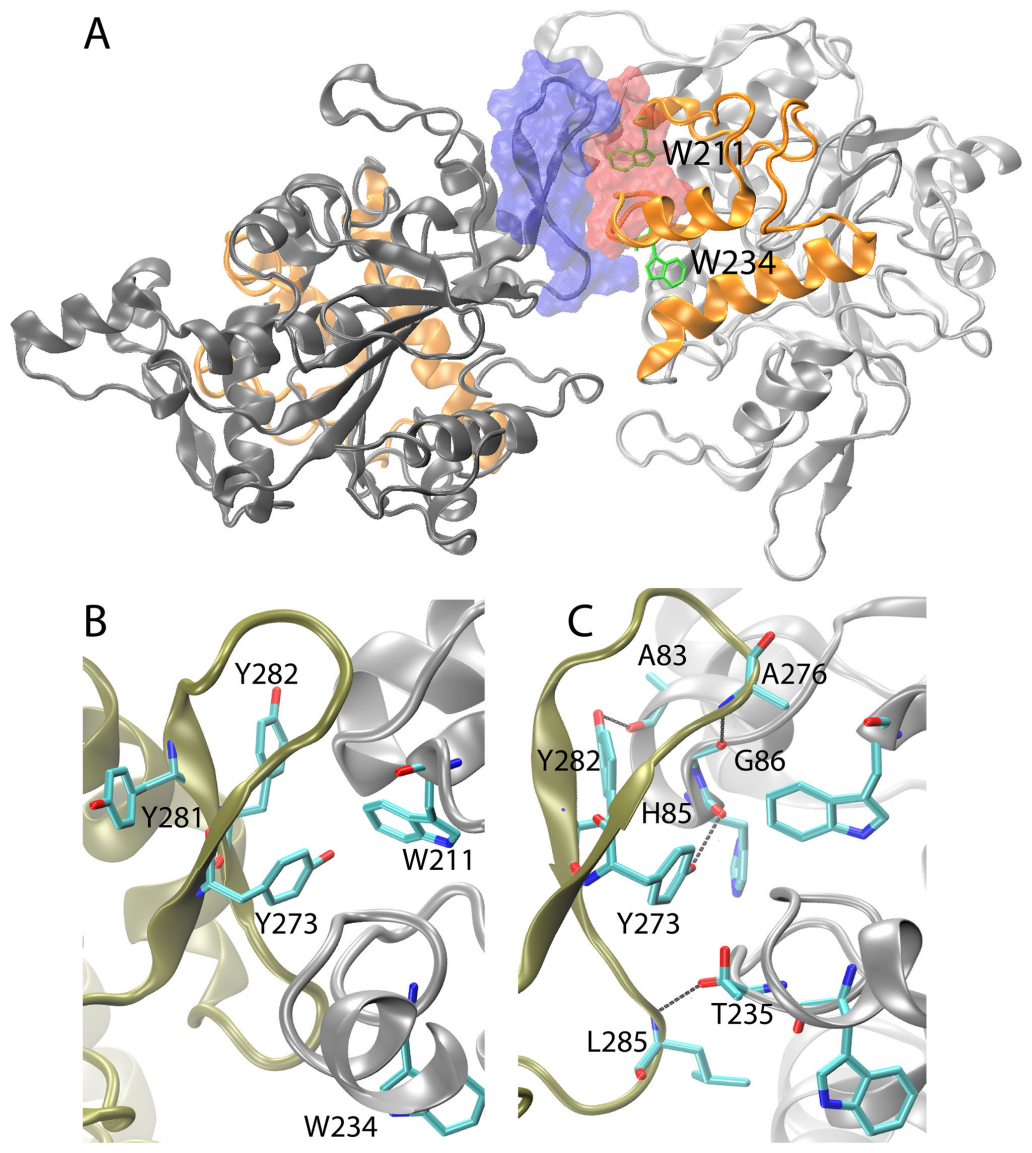
Crystal structure of the dimer BTL2. Two subunits, chain A and B are shown in black and silver, respectively, where the lids are colored in orange for both. The subunit interface is rendered by van der Waals surface: blue for chain A and *red* for chain B. The lid tryptophans W211 and W234 are shown in green sticks. (B) The aromatic cluster and (C) the network of hydrogen bonds were shown in stick models (C: cyan, N: blue, O: red).

Figure 5 illustrates a dimer structure (PDB ID: 1KU0) of a thermoalkalophilic lipase (L1) that is composed of two monomers in the closed conformation. Upon investigation of the subunit interface, an aromatic cluster of three residues is located (Figure 5B). Two of them are tyrosine residues (Y273, Y282) that were found in the anti-parallel beta-sheets of chain A, while the last one is the lid tryptophan W211 that found in a 3_10_ helix in chain B (Figure 5B). In and around this aromatic cluster, a network of hydrogen bonding was linking the monomer structures with each other (Figure 5C). Explicitly, the monomers were attached via i) a single hydrogen bond (L285-T235) at the lower section of the interface ii) three hydrogen bonds (A276-G86, Y282-G83, Y273-H85) at the upper section (Figure 5C). In these hydrogen bonds, all of the hydrogen acceptors (T235, G86, A83, H85) are backbone carbonyl oxygen atoms in chain B, while the donors are received from either the side chain hydroxyl groups (Y273, Y282) or from the main chain amide groups (L285, A286) in chain A. The aromatic interactions within the cluster and the hydrogen bonding were embedded in a way that two of the tyrosines from the aromatic cluster have involved in formation of the two hydrogen bonds with the backbone carbonyl oxygens of the other monomer. At the subunit interface we did not any observe other contacts than these hydrogen bonding and aromatic interactions. Therefore we focused on the aromatic cluster and the hydrogen bond network at the subunit interface to assess impacts of W211A on the stability of dimer. 

### Molecular dynamics simulations of the thermoalkalophilic lipase dimer

Explicit solvent MD simulations were used to assess the molecular impacts of temperature and W211A mutation on the dimer stability. Throughout the 10 ns of MD simulations, dimer stability was assessed by the occupancy of the four hydrogen bonds (Table 2). In addition to H-bond occupancies, the distributions of the H-bond distances were shown in Figure S8. Regardless of simulation temperature, the wild-type dimer was held together by four hydrogen bonds, but the total occupancy of the hydrogen bonds was lowered at high temperatures (Table 2), indicating the negative effects of high temperature on the dimer stability. The occupancy of backbone hydrogen bonds (L285-T235, A276-G86) in the wild-type dimer was not significantly affected at high temperatures. However, the hydrogen bonds formed by Y282-A83 and Y273-H85 became gradually less stable with the increasing temperature from 25°C to 125°C (Table 2). These particular observations suggested that the acute response of the dimer interactions to high temperature was received from the hydrogen bonds formed by tyrosines in the aromatic cluster, rather than the backbone hydrogen bonds. 

According to the mutant simulations, we observed less persistent hydrogen bonds at the interface than wild-type dimer regardless of temperature, implying a negative contribution of the W211A substitution to the intermolecular interactions. Particularly, W211A mutation affected the backbone hydrogen bond A276-G86 most, which was followed by the tyrosine holding hydrogen bond Y273-H85 (Table 2). Figure 5C illustrates that W211 was closely located to the upper contact point of the monomer where A276-G86, Y273-H85 and Y282-A83 pairs were found, while the lower contact point that composed of only L285-T235 pair was not close to W211. Hence, it was expected that W211A would have a more drastic impact on the upper contact point where three hydrogen bonds lied. In line with this expectation, these three hydrogen bonds (A276-G86, Y273-H85 and Y282-A83) at the upper contact point were less stable than wild-type regardless of temperature, while the backbone hydrogen bond at the lower contact point (L285-T235) was not affected at all by W211A mutation (Table 2). When the simulation temperature increased from 25°C to 125°C, the mutant W211A showed gradual destabilization in the tyrosine holding bonds (Y273-H85, Y282-A83), similar to wild-type (Table 2). However, these tyrosine holding H-bonds were much less stable at 125°C than they were in wild-type (Table 2). Moreover, contrary to wild-type simulations, the backbone hydrogen bond formed by A276-G86 was almost completely destabilized at 125°C in this mutant, while this H-bond has not been affected at all at 125°C in the wild-type. Overall, the differences in the hydrogen bond occupancies suggested a conformational change in the subunit interface led by W211A mutation, and a more drastic change for the combination of W211A mutation and high temperature. 

**Table 2:** Percent occupancy of the hydrogen bonds at subunit interface.

**Pairs**	**BTL2**			**W211A**		
**(Donor-Acceptor)**	**25°C**	**75°C**	**125°C**	**25°C**	**75°C**	**125°C**
L285-T235	64±6	56±5	38±12	63±0	52±1	38±10
Y282-A83	36±2	31±5	26±3	30±5	24±4	18±5
Y273-H85	35±6	27±3	26±1	18±6	16±8	12±3
A276-G86	21±9	17±1	22±6	9±4	5±7	3±0

All of the donors (chain A) and the acceptors (chain B) are received from backbone atoms expect from Y273, Y282 which donate their hydroxyl side chain. The mean occupancy values and errors were calculated from 10 ns of MD trajectories that repeated twice.

### Determination of the dimer stability by FoldX

The impacts of the lid tryptophan and the aromatic cluster (Y273, Y282 and W211) on the dimer stability were analyzed by FoldX using the crystal structure (PDB ID: 1KU0). The results agreed that the dimer stability was affected negatively in the case of alanine substitutions for Y273, Y282 and W211 (Table 3). The stability changes were insignificant in the case of complementary aromatic hydrophobic substitutions (W, F or Y) of the cluster residues (Table 3 and S1). Furthermore, the most drastic change in stability was observed when the aromatic cluster is deleted in the triple mutant (Y273A/Y282A/W211A) such that it reduced the interaction energy more than 10 kcal/mol. Overall, the FoldX results confirmed that the lid tryptophan W211 and the aromatic cluster is critical to stability of the intermolecular interactions at the subunit interface. 

**Table 3:** FoldX Results.

	**ΔΔG (kcal/mol)**	
**Mutation**	**Dimer**	**Monomer**
W212A	3.7	3.5
W212F	-0.3	0.5
W212Y	-0.4	1.0
Y273A/Y282A/W211A	12.6	

## Discussion

### Temperature-mediated aggregation in thermoalkalophilic lipases: a strategy for thermostability

The aggregation behavior of the thermoalkalophilic lipase BTL2 has been well studied, and it is known that that the aggregation tendency is increased at low temperatures [18,19]. To assess the impact of temperature on aggregation of BTL2 and mutants, we carried out ANS binding assays. Otherwise, DLS which is a non-invasive and reliable technique for determining the globular protein size and aggregation behavior [51], may not be practical for measurements at different temperatures [52]. ANS and protein-protein interactions as in aggregation are likely to compete over binding to the same regions in the lipase surface, which makes ANS particularly suitable for probing aggregation tendency [53,54]. Although it is not possible to comment on the oligomeric form of BTL2 by only considering the ANS spectra, as to whether it is composed of monomers or dimers, increased ANS fluorescence may reflect an increased binding surface for ANS. Hence, the ANS binding profile may be used to compare mutants with BTL2, by means of their available surface for ANS binding, and consequent aggregation tendency. 

ANS binding at 65°C was higher for BTL2 and W234A in the presence of 2-propanol, relative to buffer-only (Figure 1), confirming that 2-propanol was effective at minimizing the aggregation tendency of BTL2 and W234A. This substantial increase in ANS binding implied that the surface of BTL2 and W234 lipases was shielded by aggregation at 65°C. Considering that aggregation would not interfere with the total catalytic activity of BTL2, this observation supports that the boost in the thermostability of BTL2 and W234A at 65°C (Figure 2B) was a consequence of aggregation. Due to accumulation of intermolecular interactions in aggregates, aggregation of BTL2 would promote thermal stability in which the aggregated BTL2 would resist higher temperatures more than does non-aggregated BTL2 (Figure 2).

Regardless of 2-propanol, when the temperature increased from 55 to 65°C, the ANS fluorescence has been increased for BTL2 and W234, implying that high temperature restricts aggregation tendency. This finding agreed with the previous observations [18,19] such that aggregation of BTL2 is regulated by temperature-switches (Figure 1). The same pattern, the direct relation between ANS fluorescence and temperature was observed from 65 to 75°C only in the absence of 2-propanol. However in the presence of 2-propanol, the ANS signal was decreased for BTL2 and unchanged for W234A at 75°C, compared with the fluorescence at 65°C. This difference would be due to the thermal denaturation of BTL2 and W234A around 72-73°C, which was determined by CD spectroscopy (Figure 4D). Owing to the increased intermolecular forces favoring aggregation in the absence of 2-propanol, the thermal denaturation of BTL2 and W234A would occur at higher temperatures than the thermal denaturation in the presence of 2-propanol that prevents aggregation. In this respect, the thermal denaturation in the presence of 2-propanol might have been interfered with ANS binding, resulting in unchanged/decreased fluorescence for BTL2 and W234A at 75°C. Overall, the temperature-mediated control of aggregation was noticeable for BTL2 and W234A from Figure 1. This particular aggregation behavior of BTL2 plays an important role in its higher thermostability and the W234A did not affect this behavior (Figure 2). 

For the W211A mutant, regardless of the addition of 2-propanol and high temperatures, we did not observe significant changes in ANS signals for every temperature point. In other words 2-propanol or temperature were not effective in changing the surface for ANS binding in the W211A mutant. Taken together with the DLS analyses showing the W211A mutant does not aggregate at concentrations where BTL2 and W234A do aggregate (Table 1), ANS binding profiles confirmed that W211A impairs temperature-mediated aggregation of BTL2. Also supported by thermostability screens, the W211A mutant resulted in a much less thermostable mutant (Figure 2), suggesting that loss of aggregation led to worsened thermostability of this mutant. Hence this lid tryptophan is considered to play role in the thermostability of BTL2.

The difference between two tryptophan mutants indicated that W211 is located in a much more critical region in the lid than W234, as far as the stabilization of intermolecular interactions is concerned. A closer look into the structures of thermoalkalophilic lipases is essential to identify the molecular machinery behind the particular findings on W211A mutant. 

#### The structural model for aggregation behavior of thermoalkalophilic lipases

The structural analyses of thermoalkalophilic lipases mark the presence of a mobile structural element, the lid. The lid hinders the active site in all of the closed structures found in protein data bank (PDB) with the IDs of 1KU0 [7], 1JI3 [55], 2DSN [9], 3UMJ [56] and opens to make the active site accessible as in the structure of PDB ID: 2W22 [31]. It has been previously reported that the aggregation of BTL2 hinders the active site [19], suggesting that formation of closed form (that is inactive form) would be facilitated by aggregation, while formation of open form (that is active form) would be inhibited. Moreover, the open structure was captured in monomeric form, while all of the closed structures are dimeric and present a subunit interface for intermolecular interactions. Considering that oligomeric structures like dimers serve as templates to study aggregation behavior [57], among the closed/open conformations, the closed structures would be beneficial for *in-silico* assessment of aggregation in thermoalkalophilic lipases. 

Selection of the correct interface is crucial for understanding the mechanism of aggregation. Upon investigation of the dimer structures belonging to thermoalkalophilic lipases in PDB, an identical subunit interface was identified for all of the structures [7,9,55,56]. This unique interface was further shown to be contoured by the asymmetric portions of the lipase monomers. To illustrate the asymmetric orientation in the dimer structure, Figure 5A shows that the lid of the one monomer is partially found at the interface, while the lid of the other monomer is completely exposed to solvent. This asymmetric orientation provides the dimer with two additional faces for higher oligomer formation, suggesting that the dimer can grow to aggregates through the unique interface shown in Figure 5A. The visual inspection of the dimer structures also showed that W211 is found at the subunit interface. Considering the experimental findings confirming that W211 involves in aggregation process of BTL2, the selection of the subunit interface in the dimer is valid for proposing that aggregation is facilitated by the subunit interface resolved in the dimer structures (Figure 5A) and essentially, the intermolecular interactions located at the subunit interface might reveal the molecular machinery behind aggregation (Figure 5B-C).

Both of the Cα atoms of the lid tryptophans are found at close proximity to the subunit interface (Figure 5A). Nevertheless the side chain of W211 points towards the interface, while the side chain of W234 points towards the protein core. Such kind of conformational differences between the side chains might be used to explain the distinct outcomes of two lid tryptophans among which the W211 induces intermolecular clustering of lipase monomers leading aggregation. (See supporting information and Figure S7 for further discussion on W234)

### The aromatic cluster at subunit interface provides thermal resistance to dimer

The MD simulations of the wild-type dimer showed that high temperature attenuated the stability of the hydrogen bonds that hold aromatic tyrosines, while the other hydrogen bonds formed by backbone atoms were not affected by temperature changes at all. This suggests a direct impact of high temperature on the dimer stability through aromatic cluster residues (Table 2). Parallel to the MD simulations, FoldX analyses showed that the aromatic cluster including the lid tryptophan W211 and two tyrosines (Y273, Y282) imparted in the stability of the dimer (Table 3), proposing that the aromatic cluster resists elevated temperatures via aromatic/hydrophobic interactions during oligomerization/aggregation of the lipase. 

So far many protein interactions have been shown to be mediated by temperature switches [7,27,28,29] and similarly in our case the intermolecular interactions during the aggregation process of thermoalkalophilic lipases were to be mediated by temperature switches that accounted for improved thermostability. Interpretation of the structural information gained from the subunit interface of thermoalkalophilic lipase dimers revealed an aromatic cluster which is embedded in a network of hydrogen bonds (Figure 5). Such kind of aromatic interactions at the subunit interfaces can be dissociated by temperature switches, because the increased kinetic energy of aromatic residues i.e. increased flexibility would obstruct such residues from forming stable interactions [30]. In line with this fact, the aromatic/hydrophobic clusters at the subunit interfaces were shown to provide resistance to oligomeric structures at elevated temperatures [58,59] and disruption of the aromatic cluster has been shown to reduce thermostability [27]. Parallel to these paradigms, we proposed that the aromatic cluster including W211 at the subunit interface of the thermoalkalophilic lipases is in charge of the reversible dissociation of the dimer/aggregates by increased kinetic energy, i.e. the temperature-mediated switch of the aggregation. Only at high temperatures the aromatic cluster gets mobilized and their increased kinetic energy perturbs the rigidity of the subunit interface preventing the aromatic interactions between monomers and aggregation. In this respect, the aromatic cluster, essentially W211, confers thermostability by stabilizing the intermolecular interactions at elevated temperatures.

### W211 stabilizes the intermolecular interactions during aggregation

**Figure 6:**
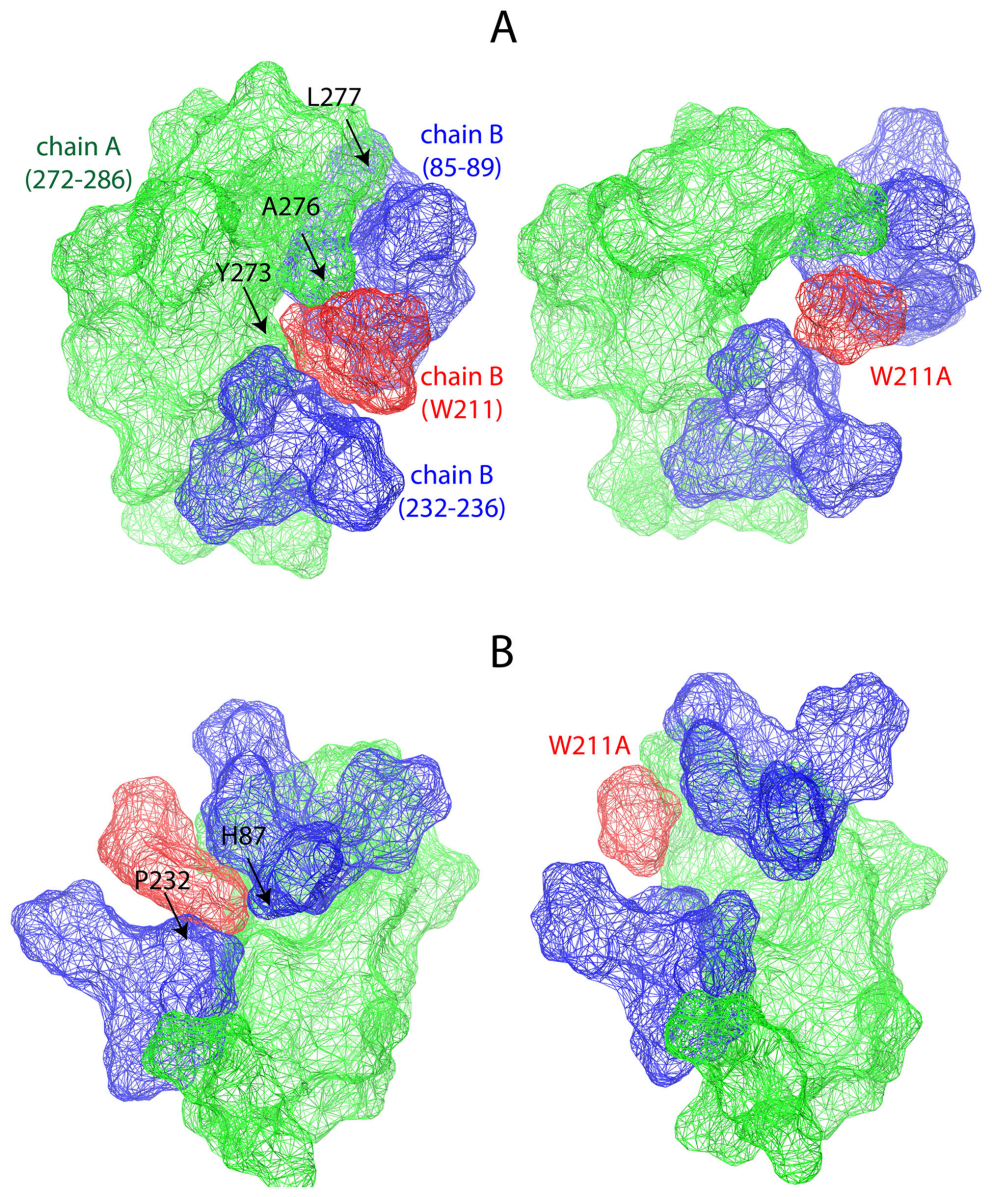
The structural impact of W211A mutation on the subunit interface. The subunit interface is rendered by van der Waals surface using the colors; green for chain A, blue for chain B, and *red* for 211 (W or A) that is found in chain B. The arrows indicate the residues that change conformation upon W211A mutation in (A) chain A and (B) chain B. The representation in (A) has been rotated 180° around the left diagonal axis to obtain (B), and the snapshots were taken from the simulations performed at 75°C.

Inspection of the native and the W211A subunit interfaces revealed the structural impact of W211 on the intermolecular interactions (Figure 6A). In the wild-type configuration, the exposed side chain of W211 serves as a binding site for the other monomer and the side chain of W211 directly interacted with Y273 and A276 of the adjacent subunit. Upon W211A mutation, three residues (Y273, A276, L277) at the subunit interface change their conformations. Two of these residues (Y273, A276) are found in the hydrogen bond network and hence, the conformational change in Y273 and A276 due to W211A mutation resulted in the disruption of the particular hydrogen bonds formed by A276-G86 and Y273-H85 (Table 2 and Figure S8).

In addition to the effects of W211A on H-bond network, deletion of W211 would essentially lead to disruption of the intermolecular aromatic interactions because the aromatic cluster is formed by receiving only W211 from one monomer, while it is received by two tyrosines in the other monomer. In line with this expectation, Y273 dissociated from the subunit interface in the W211A mutation (Figure 6A), leading to disruption of the aromatic interactions between monomers. At a distant proximity, the W211A mutation also affected the conformation of L277 at the subunit interface. In the wild-type L277 interacted with the other monomer while in the W211A mutant the conformation of L277 was altered and its interactions were lost (Figure 6A). Overall, the W211A mutation leads to destabilization of the dimer by disrupting the aromatic cluster and by reducing hydrogen bond occupancies regardless of temperature (Figure 6A and Table 2).

### W211 stabilizes the intramolecular interactions at high temperatures for catalytic activity

Previously, BTL2 heterologously expressed in *Pichia pastoris*, has been shown to work optimally at 65°C [60]. Our result was slightly lower than that since the BTL2 showed its maximum activity at 62°C (Figure S4). This slight difference could be due to the expression system used in our study that is *E. coli*, different from what was used in the previous study [60]. 

We performed thermoactivity assays where the real-time lipase activity is quantified to determine *T*_*opt*_ of lipases. Thermoalkalophilic lipases have two distinct lid conformations that are open and closed corresponding to the active and the inactive lipase respectively. The lipase becomes active and attains open conformation via physical attachment of its lid to substrate interface, and in the absence of substrate it tends to stay inactive in closed form [61]. Considering these facts, structural insights regarding thermoactivity could be obtained from the open-conformation when the lipase is captured in active form.

**Figure 7:**
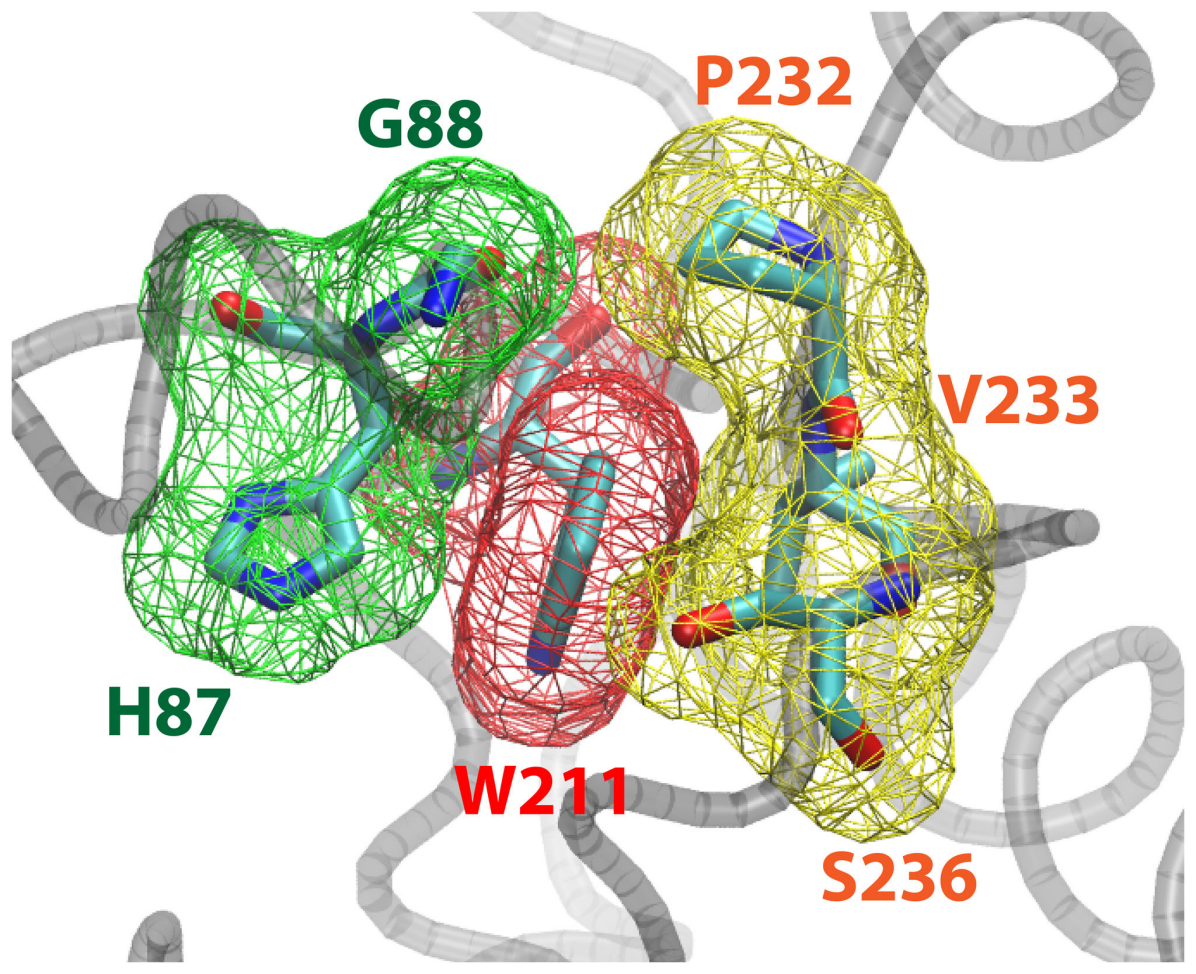
W211 in the active conformation. The domain formed by W211 in the open-active conformation (PDB ID: 2W22) is rendered by van der Waals surface. H87-G88 (green) and P232-V233-S236 (yellow) tightly packs the side chain of W211 colored in (red).

Thermoactivity assays showed that the W211A significantly decreased the *T*_*opt*_ of BTL2 (Figure 3). To visualize this particular impact of W211, we inspected the open conformation of the thermoalkalophilic lipase (PDB ID: 2w22) and we found the side chain of W211 completely buried by two regions (H87-G88 and P232-V233-S236) (Figure 7). The tight packing of W211 side chain shows the involvement of W211 in intramolecular interactions in the open-form. The contribution of W211 to the stability is quantified by FoldX and the results agreed that W211A decreased the stability of open-monomer significantly with 3.5 kcal/mol. Similarly tyrosine (1.0 kcal/mol) and phenylalanine (0.5 kcal/mol) substitutions, although less than the alanine substitution, decreased stability (Table 3). We further inspected the W211 in the closed structures that have been employed in the MD simulations. Figure 6B illustrated the impact of W211A mutation in the closed monomer. Particularly, the W211A substitution changed the conformations of two aromatic residues: H87 and P232 (Figure 6B), resulting in the disruption of the particular intramolecular interactions. Overall, these investigations suggest that W211 is an essential residue for the intramolecular interactions in the lipase monomer irrespective of its conformation, closed or open. Considering the lower *T*_*opt*_ of the W211A mutant than BTL2, we suggest that the lid tryptophan W211 also plays an important role in stabilization of the open monomer at elevated temperatures. 

In general, thermostability could be related to thermoactivity [62], nonetheless it cannot guarantee catalytic activity at high temperatures i.e. thermoactivity, particularly when a distinct structural domain, other than catalytic domain, is responsible for thermostability [17]. In our case, we observed for the W211A mutant that it affected the thermostability (Figure 2) and thermoactivity of BTL2 (Figure 3), suggesting that apart from being involved in the stabilization of the dimer (Table 2 and 3), W211 is also crucial for the monomer stability (Table 3). Therefore, we report that the conserved the lid tryptophan acts as a hub residue to confer stability to the aggregated and the active form of BTL2 at elevated temperatures.

## Conclusions

We attempted to identify the role of aggregation in the thermostability of BTL2 and found that aggregation contributes to thermostability through an aromatic cluster and a hydrogen bond network at the subunit interface of the dimeric lipase. Because the experimental results on BTL2 would be valid for the rest of the thermoalkalophilic lipases owing to 90% of sequence homology among them, we highlight that aggregation would be a characteristic thermostability strategy of the thermoalkalophilic lipases. Particularly, the experimental findings on W211, a member of the aromatic cluster at the subunit interface, supported that aggregation contributes to thermostability because W211A blocked aggregation and resulted in a much less thermotolerant variant of BTL2. Besides the fact that that W211 is an essential residue for the improvement of thermostability via aggregation, it is also required for thermoactivity, implying its contribution to intramolecular interactions as well. 

Aggregation of industrial enzymes is a major concern, in which it decreases the solubility of the recombinant protein products and thus results in the loss of yield in biocatalysis. The last point to be noted on this mutation is that the decrease in thermoactivity and thermostability of the W211A mutant may not necessarily be a disadvantage, since the W211A mutant will represent a promising mutation that increases the solubility of BTL2. Hence, we conclude that identification of the lid tryptophan W211 provided an understanding of how thermoalkalophilic lipases maintain stability and activity at high temperatures and also insights to design of lipase analogues for industrial use.

## Supporting Information

Figure S1 **SDS-PAGE results of expression and purification of wild-type and mutant lipase.** (Left) Lane 1: Molecular weight marker (Fermentas #SM0671); 2, 4, 6: soluble fractions from BTL2, W211A and W234A samples, respectively and 3, 5, 7: batch purifications using nickel coated beads. (Right) The elution fractions from column purification analyses; top gel: W211A; bottom-left: BTL2 and bottom-right: W234A. The predicted molecular weight for the recombinant lipases (43 kDa) falls in between the reference protein bands of 55 and 35 kDa.
(TIF)

Figure S2 **DLS results of three lipases of three measurements showing the mean intensity and mean volume as a logarithmic function of the hydrodynamic radius.**
(TIF)

Figure S3 **Thermostability of BTL2 at different incubation concentrations.**
(TIF)

Figure S4 **Thermoactivity of BTL2 and W234A.**
(TIF)

Figure S5 **The RMSD analyses of molecular dynamics simulations.**
(TIF)

Figure S6 **The RMSF analyses of Cα atoms of the backbone atoms; wild-type and W211A mutant dimer systems correspond to a total of 388 residues per monomer.** Average values of two simulations were plotted.
(TIF)

Figure S7 **The lid region and the domain formed by W211 is shown for the closed-inactive (PDB ID:1KU0_A) and open-active (PDB ID: 2W22) conformations.** The domain formed by W211 is presented in van der Waals surface and the lid is in tubes. The other lid tryptophan W234 is also shown in sticks model. The red arrows show the conformational changes in the open-active form with respect to closed-inactive form.
(TIF)

Figure S8 **Distributions of H-bond distances at the subunit interface.** The same legend applies to all figures. The distributions are collected from 10 ns of trajectories in 500 windows.
(TIF)

Text S1 (DOCX)

Table S1 **FoldX Results.**
(XLSX)
