# Supplementary figures and images for: The Conserved Lid Tryptophan, W211, Potentiates Thermostability and Thermoactivity in Bacterial Thermoalkalophilic Lipases
Source: PLoS One. 2013 Dec 31;8(12):e85186. doi: 10.1371/journal.pone.0085186 (PMC3877348; doi:10.1371/journal.pone.0085186)

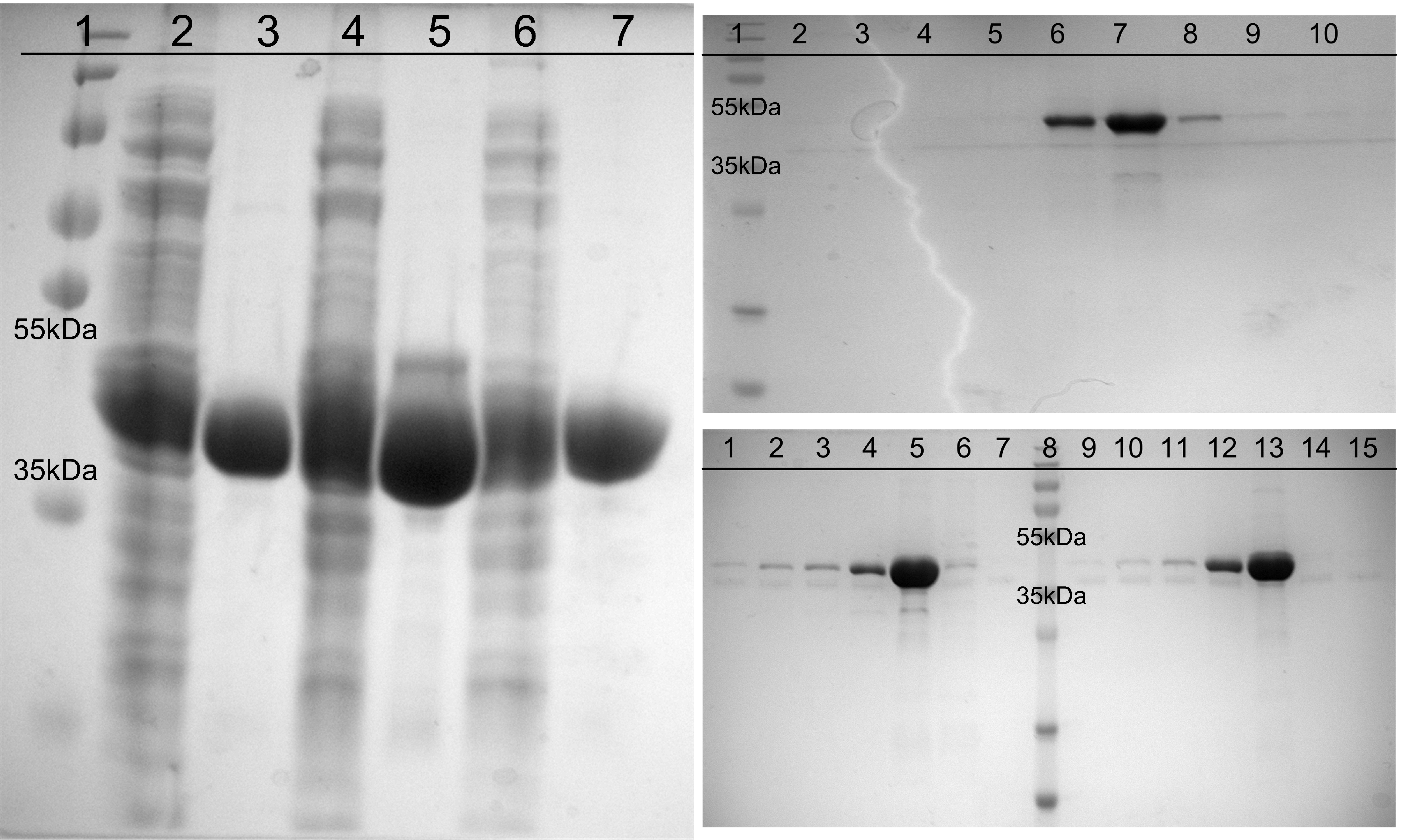

Supplement: Figure S1 — SDS-PAGE results of expression and purification of wild-type and mutant lipase. (Left) Lane 1: Molecular weight marker (Fermentas #SM0671); 2, 4, 6: soluble fractions from BTL2, W211A and W234A samples, respectively and 3, 5, 7: batch purifications using nickel coated beads. (Right) The elution fractions from column purification analyses; top gel: W211A; bottom-left: BTL2 and bottom-right: W234A. The predicted molecular weight for the recombinant lipases (43 kDa) falls in between the reference protein bands of 55 and 35 kDa. (TIF) [file pone.0085186.s001.tif]

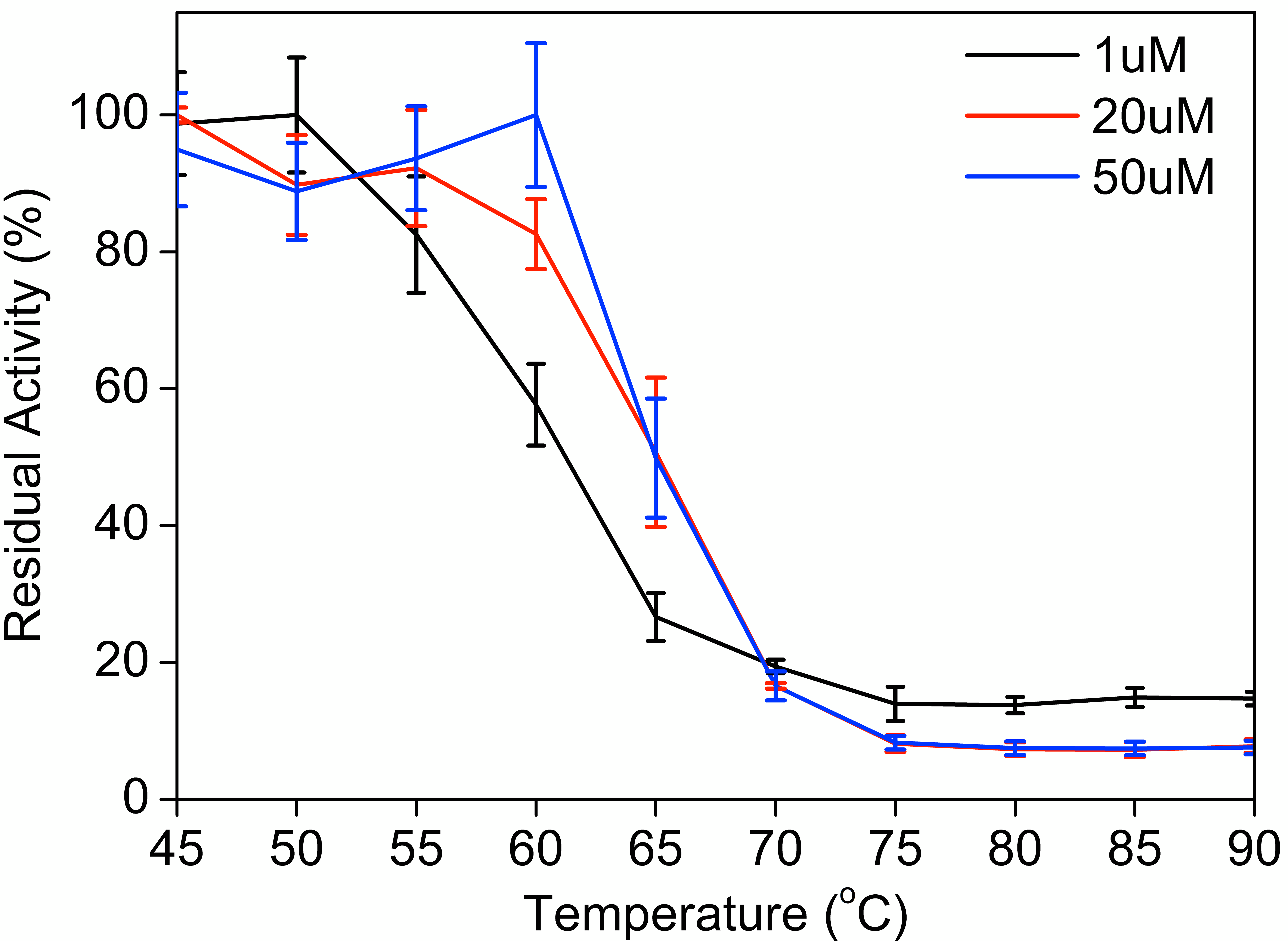

Supplement: Figure S3 — Thermostability of BTL2 at different incubation concentrations. (TIF) [file pone.0085186.s003.tif]

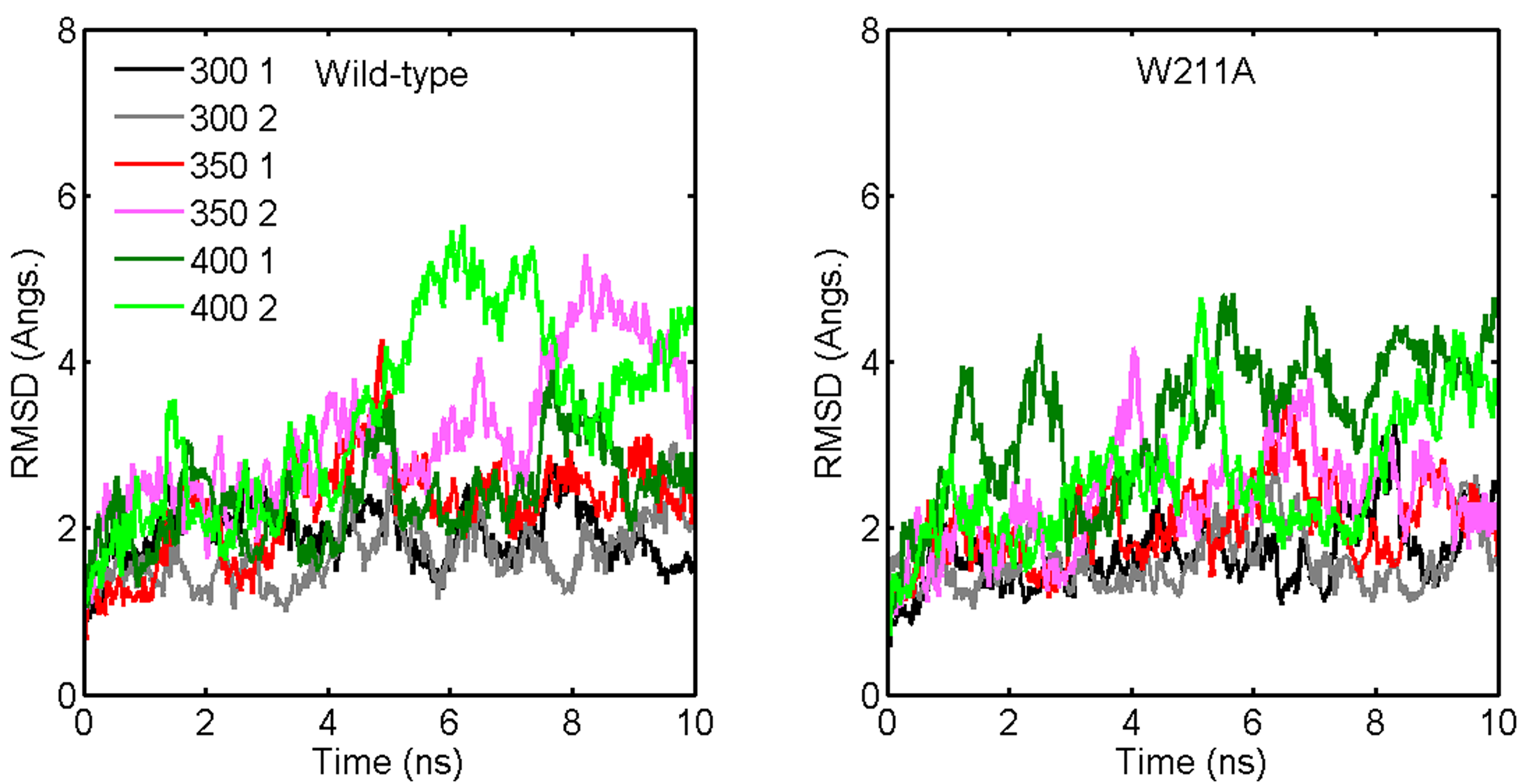

Supplement: Figure S5 — The RMSD analyses of molecular dynamics simulations. (TIF) [file pone.0085186.s005.tif]

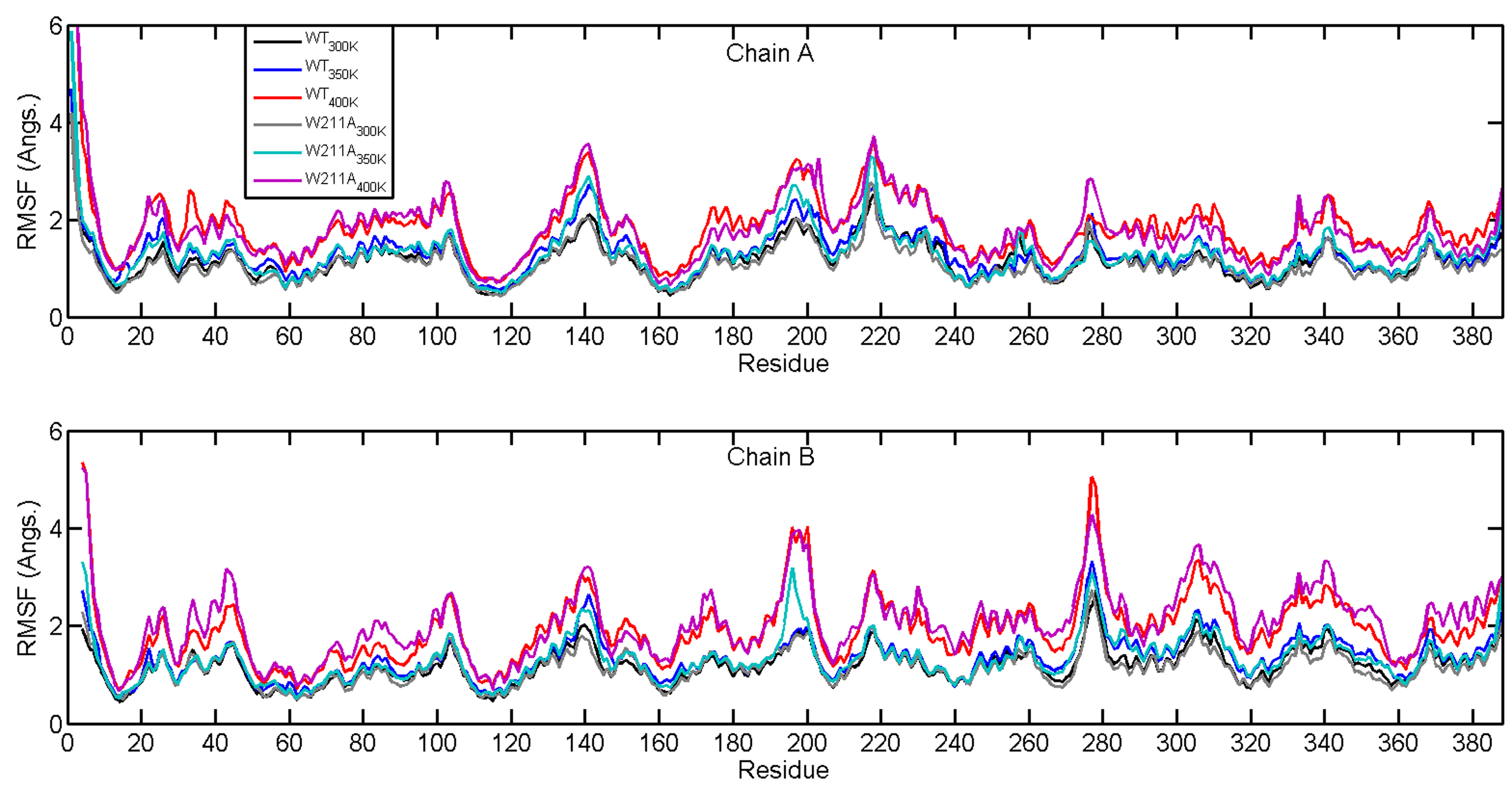

Supplement: Figure S6 — The RMSF analyses of Cα atoms of the backbone atoms; wild-type and W211A mutant dimer systems correspond to a total of 388 residues per monomer. Average values of two simulations were plotted. (TIF) [file pone.0085186.s006.tif]

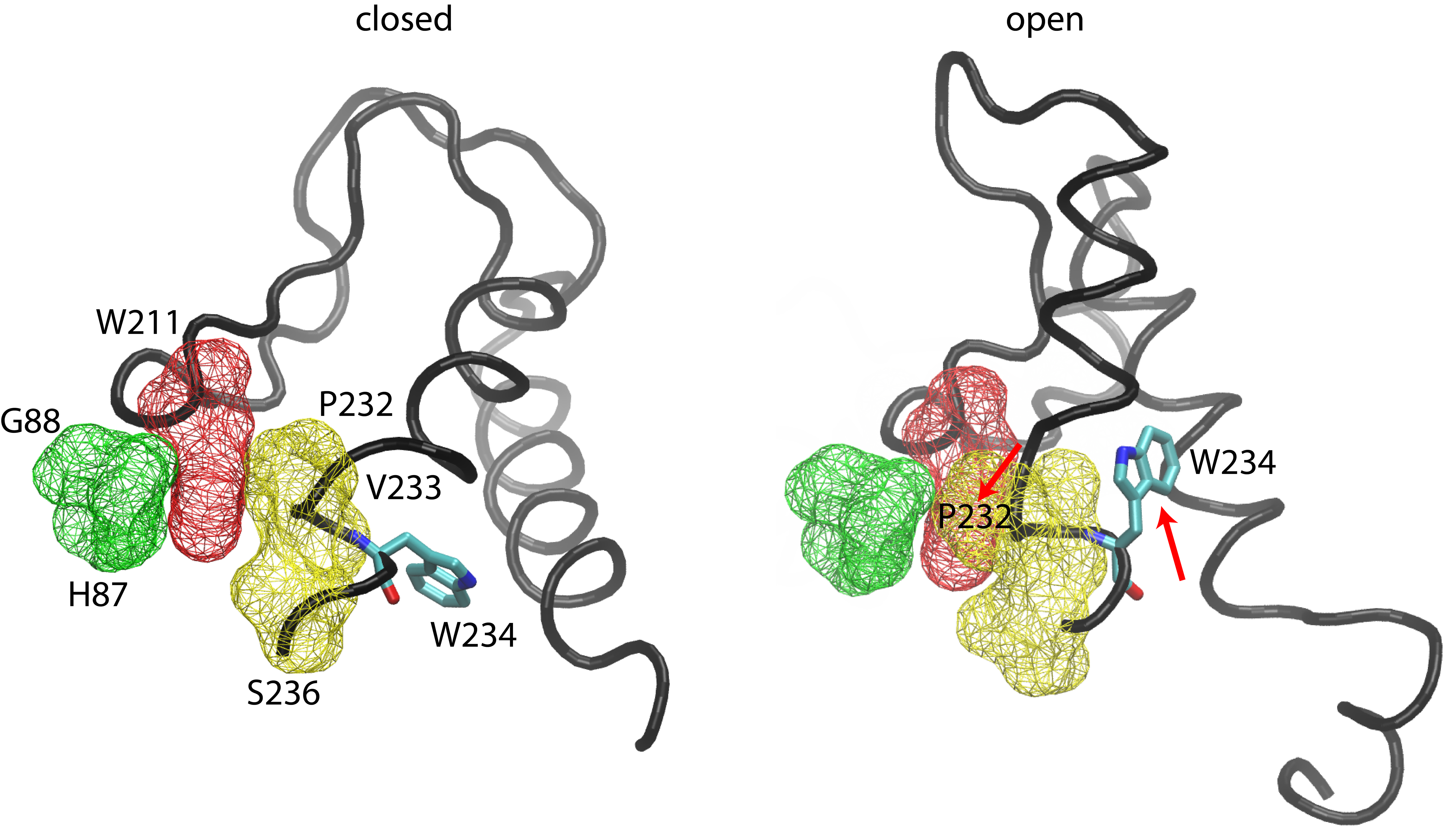

Supplement: Figure S7 — The lid region and the domain formed by W211 is shown for the closed-inactive (PDB ID:1KU0_A) and open-active (PDB ID: 2W22) conformations. The domain formed by W211 is presented in van der Waals surface and the lid is in tubes. The other lid tryptophan W234 is also shown in sticks model. The red arrows show the conformational changes in the open-active form with respect to closed-inactive form. (TIF) [file pone.0085186.s007.tif]

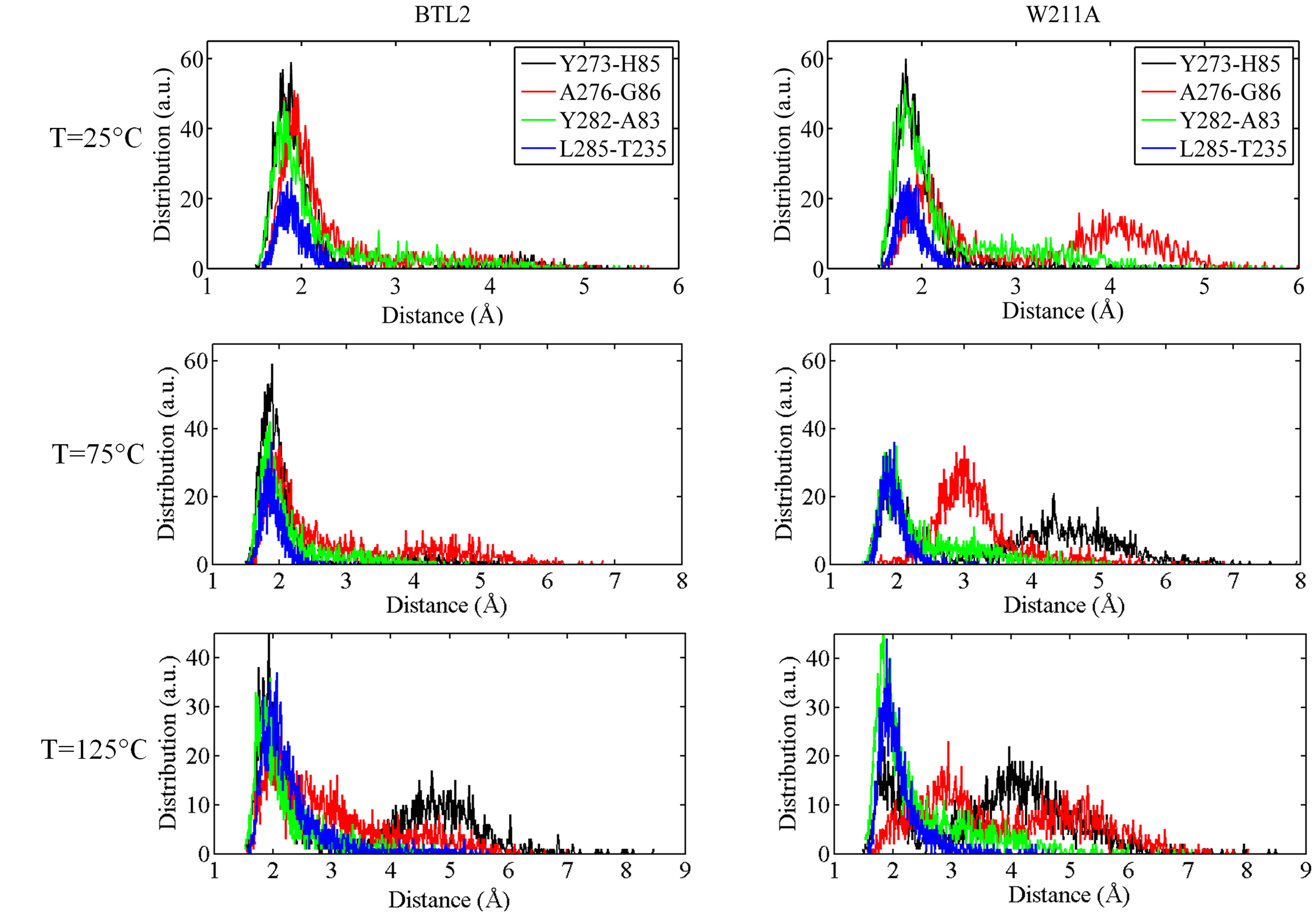

Supplement: Figure S8 — Distributions of H-bond distances at the subunit interface. The same legend applies to all figures. The distributions are collected from 10 ns of trajectories in 500 windows. (TIF) [file pone.0085186.s008.tif]
