# Supplementary material for: The Conserved Lid Tryptophan, W211, Potentiates Thermostability and Thermoactivity in Bacterial Thermoalkalophilic Lipases
Source: PLoS One. 2013 Dec 31;8(12):e85186. doi: 10.1371/journal.pone.0085186 (PMC3877348; doi:10.1371/journal.pone.0085186)
Supplement: Text S1 — (DOCX) [file pone.0085186.s009.docx]

**SUPPORTING TEXT**

**The RMSD and RMSF analyses of MD simulations**

The wild-type and the mutant W211A dimer systems were simulated for 10 ns at 300 K, 350 K and 400 K. The backbone atoms (C, N, Cα) were found to be moved within 0.5 Å at 300 K for both dimer structures while this distance have risen to 2 Å at 350 K and 400 K (Fig. S5). Overall, we did not see any differences in the RMSD of the protein backbone upon W211A mutation and also reported that the simulations showed a rough equilibration.

We used the dimer structure (PDB ID: 1KU0) in MD simulations to assess the impacts of temperature on the intermolecular interactions at the subunit interface. Particularly, at 400 K we observed higher flexibility in certain regions from both chain (residues from 275-to-280) A and chain B (80-to-85) than that at 300 or 350 K. The residues from 275-to-280 in chain A was less flexible than those in chain B, while the residues from 80-to-85 in chain B was less flexible than those in chain A (Fig. S6). These particular portions of the monomers that showed increased mobility at high temperatures were the subunit interface residues, suggesting that the residues found at the interface were more rigid with respect to the condition that they are exposed to solvent. This also concurs with the idea that oligomerization and/or aggregation enhance the stability of the lipase structure by reducing the flexibility of the subunit interface.

**Structural insights from the comparison of open-monomer with closed-monomer**

To visualize the impact of W211 on the intramolecular interactions, we inspected the monomer thermoalkalophilic lipase structures: open monomer (PDB ID: 2w22) and closed monomer (PDB ID: 1KU0 -chain A) (Fig. S7). The lid region which changes its conformation between closed and open states [*Methods Enzymol* **1997**, *284*, 3] was colored in orange and the lid tryptophans were also shown in both of the structures. Despite that W211 is in the lid region, it does not change its conformation such that it is found in a 3_10_ helix for both of the closed and open states. For both of the monomers, the side chain of W211 is tightly packed by two distinct regions (residues from 85-to-89 and 232-to-236). Additionally, the W211 side chain was completely buried by either the other monomer (Fig. S7A), or one of the lid helices (Fig. S7B) in the closed dimer and open monomer structures, respectively.

It was observed from MD simulations that the residues from 190-to-200 in chain B were more flexible in the W211A mutant than the wild-type dimer (Fig. S5). The flexibility of the same region was previously found to be involved in lipase activation [*JBC* **2002**, *19*, 17041]. The enhanced fluctuations upon W211A mutation would imply that lipase activation is initiated at lower temperatures in case of W211A.

According to the experimental analyses of W234A, we concluded that this residue is not critical for aggregation, thermostability or thermoactivity of BTL2. Fig. S7 illustrates that, however W234 is located fairly close to the subunit interface, its side chain points towards the protein core. Moreover, in the open monomer the side chain of W234 changes conformation with respect to the closed form (Fig. S7), such that W234 side chain is exposed to solvent in the active conformation, while it was buried in the closed conformation. These observations could support that W234 cannot form any interactions with the other monomer, as W211 can. Yet, W234 may be engaged in binding to the substrate interface and be important for interfacial activation by exposing its hydrophobic side chain towards substrate-interface.
